# Robot-supported upper limb training in a virtual learning environment : a pilot randomized controlled trial in persons with MS

**DOI:** 10.1186/s12984-015-0043-3

**Published:** 2015-07-23

**Authors:** Peter Feys, Karin Coninx, Lore Kerkhofs, Tom De Weyer, Veronik Truyens, Anneleen Maris, Ilse Lamers

**Affiliations:** REVAL Rehabilitation Research Center, BIOMED Biomedical Research Institute, Faculty of Medicine and Life Sciences, Hasselt University, Martelarenlaan 42, 3590 Diepenbeek, Belgium; Expertise Centre for Digital Media - tUL – iMinds, Hasselt University, Wetenschapspark 2, 3590 Diepenbeek, Belgium; Rehabilitation and MS Center Overpelt, 3900 Overpelt, Belgium

## Abstract

**Background:**

Despite the functional impact of upper limb dysfunction in multiple sclerosis (MS), effects of intensive exercise programs and specifically robot-supported training have been rarely investigated in persons with advanced MS.

**Aim:**

To investigate the effects of additional robot-supported upper limb training in persons with MS compared to conventional treatment only.

**Methods:**

Seventeen persons with MS (pwMS) (median Expanded Disability Status Scale of 8, range 3.5–8.5) were included in a pilot RCT comparing the effects of additional robot-supported training to conventional treatment only. Additional training consisted of 3 weekly sessions of 30 min interacting with the HapticMaster robot within an individualised virtual learning environment (I-TRAVLE). Clinical measures at body function (Hand grip strength, Motricity Index, Fugl-Meyer) and activity (Action Research Arm test, Motor Activity Log) level were administered before and after an intervention period of 8 weeks. The intervention group were also evaluated on robot-mediated movement tasks in three dimensions, providing active range of motion, movement duration and speed and hand-path ratio as indication of movement efficiency in the spatial domain. Non-parametric statistics were applied.

**Results:**

PwMS commented favourably on the robot-supported virtual learning environment and reported functional training effects in daily life. Movement tasks in three dimensions, measured with the robot, were performed in less time and for the transporting and reaching movement tasks more efficiently. There were however no significant changes for any clinical measure in neither intervention nor control group although observational analyses of the included cases indicated large improvements on the Fugl-Meyer in persons with more marked upper limb dysfunction.

**Conclusion:**

Robot-supported training lead to more efficient movement execution which was however, on group level, not reflected by significant changes on standard clinical tests. Persons with more marked upper limb dysfunction may benefit most from additional robot-supported training, but larger studies are needed.

**Trial registration:**

This trial is registered within the registry Clinical Trials GOV (NCT02257606).

## Background

Multiple Sclerosis (MS) is a chronic progressive neurologic disease affecting young adults, manifesting with multiple neurological dysfunctions in the motor, sensory, visual and cognitive systems. Motor symptoms such as muscle weakness, incoordination and hypertonia affecting balance, walking and upper limb function occur frequently. It is known that many persons with MS (pwMS) show reduced physical activity which contributes to marked functional limitations [1, 2]. In the recent decade, numerous studies in MS have demonstrated that exercise therapy can have beneficial effects on different levels of the International Classification of Functioning (ICF) [3–5]. Physical exercise interventions in these studies were mostly targeted towards the lower limb muscle function, balance and/or walking [4, 5], the latter being perceived as a valuable bodily function which is already affected at early disease onset [6]. However, as the disease progresses, the upper limbs get more affected which leads to accumulated disability especially when dysfunction is present bilaterally. Kierkegaard et al. reported that 76 % of a large Swedish sample of pwMS showed at least some disability regarding manual dexterity, leading to a significant negative impact on the performance of activities of daily life in half of all pwMS [7], as a result reducing personal independence and quality of life [8].

A systematic literature search on conventional motor training programs for the upper limbs in MS revealed that only a limited number of studies were exclusively dedicated to improve upper limb function [9]. In fact, many studies investigated primarily the effects of multidisciplinary treatment or exercise therapy for the total body in pwMS without considerable upper limb dysfunction [10–13]. Positively, the results suggest a restorative potential of the upper limb function in pwMS. However, more research is needed in severely disabled pwMS. In a more advanced stage of the disease with severe disability, there is an increasing multiplicity of symptoms in MS and related bodily functioning problems (for example gait and balance dysfunction), and thus reducing time allocated to upper limb treatment. This lack of therapy time is in contrast with the knowledge that training volume and intensity is important to achieve improvements. In this framework, robot-supported interventions for the upper limb have been increasingly applied given its advantage of high intensity training, volume and duration which can be delivered without constant presence of a therapist. In both acute and chronic stroke patients, randomized controlled trials and systematic reviews concluded that robot-supported rehabilitation was as effective as intensive comparative conventional treatment for improving motor function, and furthermore, potentially activity level [14, 15]. In MS, uncontrolled pilot studies in small sample sizes (<10) have indicated the potential of robot-supported upper limb training in pwMS with muscle weakness and cerebellar symptoms. Gijbels et al. (2011) reported, in highly disabled and wheelchair bound pwMS (Expanded Disability Status Scale (EDSS): 7–8.5), beneficial effects of an additional 8 week upper limb training with a 3D electromechanical exoskeleton providing anti-gravity support (ARMEO Spring, Hocoma, CH) [16, 17]. The usefulness of a 2D end-effector robot (Braccio di Ferro) has been repeatedly reported for assessing and training upper limb motor co-ordination [18, 19]. After 8 training sessions of 1 h over 2–4 weeks with this device, pwMS (EDSS 3.0–6.5) showed gains in velocity, linearity and smoothness of reaching movements as well as decrease in ataxia and tremor scores. The latter results were replicated recently in a RCT [20]. Above-mentioned studies indicate that robot-supported upper limb training is potentially effective to improve motor function, but knowledge on impact on activity level is sparse. It is concluded that controlled trials with a comprehensive test battery on different ICF levels is warranted.

To further enhance the applicability and effectiveness of technology-supported training modalities, virtual reality applications are increasingly developing, with recent reviews indicating benefits on upper limb activity level in persons with stroke [21, 22]. In MS, the use of serious gaming in combination with off-the-shelf technology such as Kinect seems enjoyable and may ameliorate rehabilitation adherence with regards to balance [23]. Only few developments are being known for the upper limb [24]. Upper limb rehabilitation in a virtual environment has the advantage that pwMS with severe dysfunctions can experience success during training by increasing motivation for intensive and long-term active motor training [25, 26]. Optimal custom-built virtual learning environment with a personalised approach that incorporate aspects of motor and cognitive-social learning are available [27].

This pilot RCT investigated the effects of an additional 3D robot-supported upper limb training of 8 weeks in disabled pwMS; incorporating a personalized virtual learning environment (see Methods section), in comparison to pwMS receiving conventional treatment only. Both movement quality during robot-mediated movement tasks as well as clinical measures at function and activity levels were assessed.

## Methods

### Participants

Seventeen pwMS diagnosed according to the McDonald criteria and upper limb weakness determined by the Motricity Index (MI; score < 85), participated. Participants with (nearly) total paralysis of both upper limbs based on the Motricity Index were excluded. This was due to the robot-supported training, because this system required the ability to produce independent movements with the upper extremity with a minimal 3D amplitude of 6 cm. Similarly, pwMS presenting with visual, cognitive and cerebellar dysfunctions, as detected by the neurologist during evaluation of the related functional systems of the EDSS, and potentially interfering with task execution, were excluded. PwMS having a relapse in the last month before study onset or receiving relapse-related glucocorticosteroid treatment were also excluded.

Participants were either hospitalised in the Rehabilitation and MS Center Overpelt (Belgium) for multiple weeks receiving treatment from multiple disciplines (for example, physiotherapy, occupational therapy, psychology, speech therapy), or bi-weekly attended rehabilitation sessions at the rehabilitation center on an ambulant basis, i.e. with the participant living in the community and coming to the center for therapy purposes. The conventional rehabilitation program consisted of 2 h multidisciplinary treatment per day including 30 min physiotherapy, 30 min occupational therapy and 60 min group physiotherapy, speech therapy or psychotherapy depending on the needs of the participant. Ethical approval was obtained from the Medical Ethical Committee of the Hasselt University and the ethical committee of the Rehabilitation and MS centre of Overpelt. All participants gave their written consent. The authors confirm that all ongoing and related trials for this intervention are registered within the register Clinical Trials GOV (NCT02257606).

### Experimental design

**Fig. 1:**
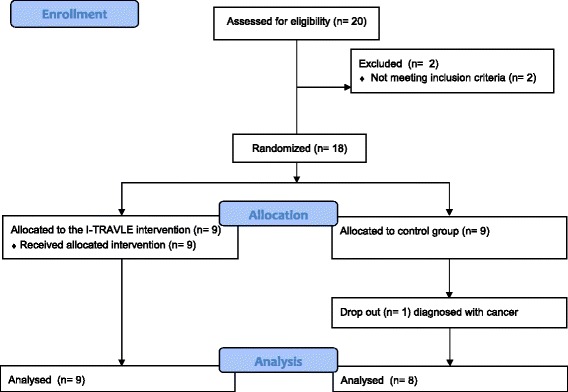
CONSORT flowchart

This study is a randomised controlled trial in pwMS differentiating a control group receiving conventional therapy, and an intervention group additionally receiving robot-supported training. Robot training was provided for the duration of 8 consecutive weeks at a frequency of 3 times per week, on Mondays, Wednesdays and Fridays. The training sessions always took place at the same time of day and lasted for 30 min. In total the participants received 12 h of robot training in addition to usual care. All completed the training between April 11th and September 30th, 2011. Measurements were performed at week 0 and week 8 to compare changes of the intervention group after the additional robot-supported therapy with the longitudinal changes of the control group receiving only 8 weeks of conventional therapy. Included pwMS were randomly allocated in an MS intervention group (*n* = 9) and a MS control group (*n* = 8) by the sealed envelope method by a person not involved in the trial (Fig. 1). Based on the MI, the weakest upper limb was identified for evaluation and training. However, in case the weakest upper limb was almost completely paralyzed, the other upper limb was trained if eligible by showing muscle weakness. When both arms were equally impaired, the participant’s preference was taken into account. In the control group, the same criteria was applied for selecting the upper limb that was evaluated.

### Experimental apparatus & procedure

**Fig. 2:**
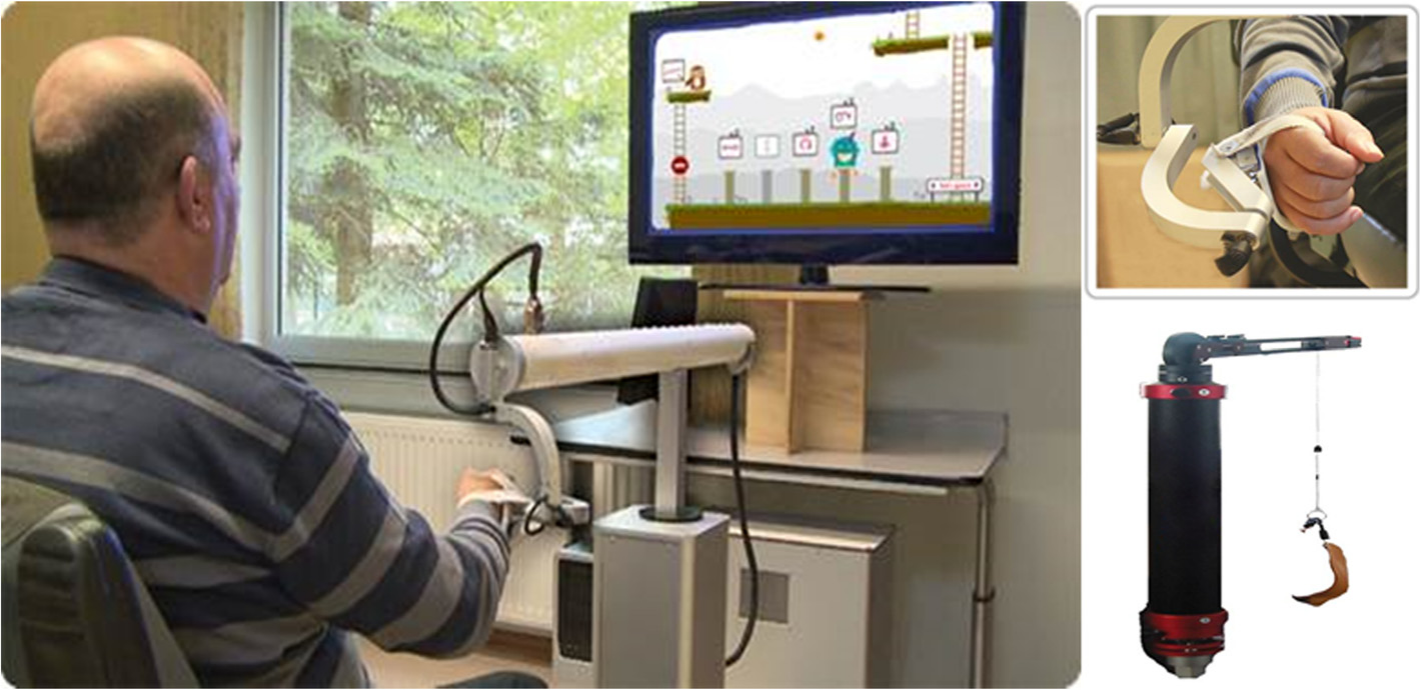
Hardware set-up, including a haptic robot, sling and visual display

Figure 2 shows an overview of the system set-up. The HapticMaster robot (MOOG, the Netherlands) functioned both as an output device, providing haptic feedback during the training by guiding or hindering movements with exerted forces, but also as an input device, allowing navigation within a virtual learning environment. The HapticMaster was chosen as it has a relatively large 3D workspace for upper limb training (36 cm for depth, 40 cm height and 1 rad for mediolateral movements). This robot has been previously used in stroke rehabilitation [28, 29]. Training occurred in a sitting position in a chair or in a wheelchair; in the case of wheelchair-bound pwMS. The position of chair or wheelchair towards the HapticMaster was standardized by the use of a numbered checkerboard placed on the floor. The participant’s hand was placed in a small palmar brace embedded in an ADL gimbal that was connected to the endpoint of the HapticMaster. Participant’s hand movements were unrestrained, allowing spontaneous opening and closing of the hand. A large 40” screen was placed at 1.5 m in front of the participant to display the virtual learning environment.

At the beginning of each session, the need for gravity compensation was individually determined using an active positioning procedure requiring a sustained endpoint position in space [30]. This procedure was performed to further allow pwMS with prominent muscle weakness to engage in intensive training, as gravity compensation facilitates the execution of sufficiently large upper limb movements [30–32]. Gravity compensation was provided by the HapticMaster at the hand, or additionally by means of a sling (FOCAL, Tilburg, the Netherlands) supporting the elbow. Then, the active workspace in three dimensions was individually determined for matching the programmed locations of targets in the virtual learning environment with the participant’s active capabilities.

**Fig. 3:**
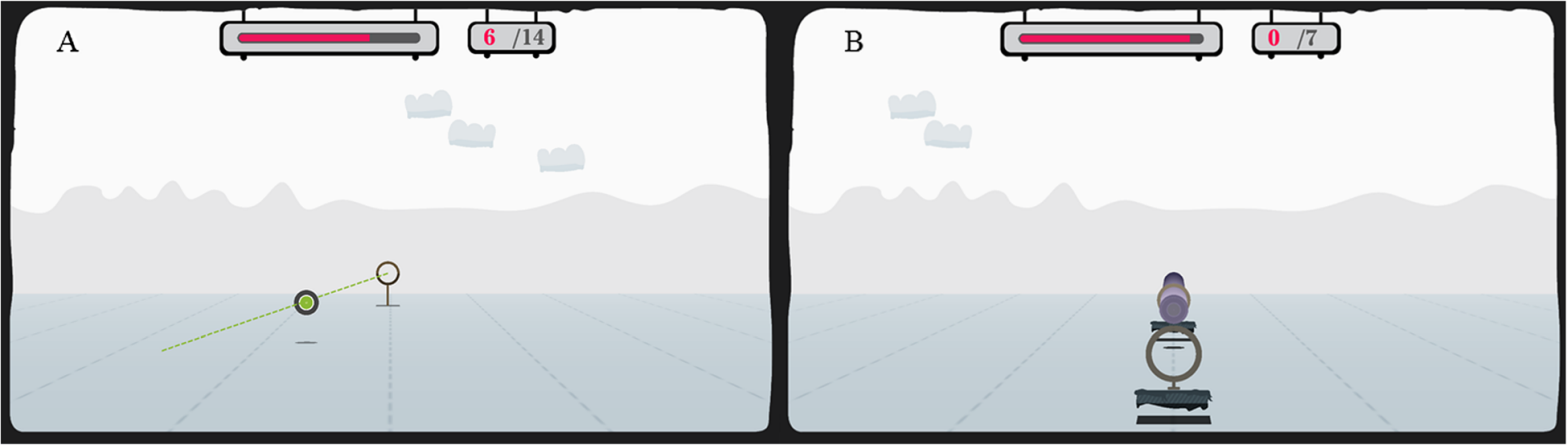
Example of basic motor function exercise ‘reach’ (**a**) and ‘pull’ (**b**). Participants have to reach or pull the disk towards the target. Visual feedback on the correctness of the executed trajectory is provided by colour changes of the disk (green, orange, red). During pulling, subjects experience resistance on, or are pulled to, the trajectory

**Fig. 4:**
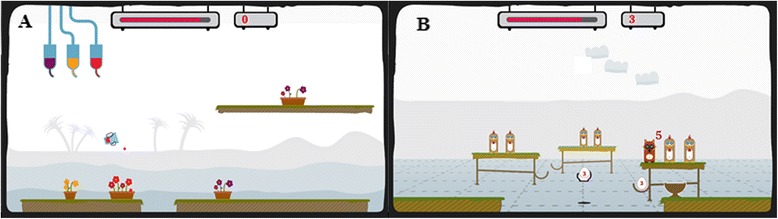
Serious games **a** ‘Watering the flowers’ and **b** ‘Chicken run’. **a** In ‘watering the flowers’, the skill components lifting, transporting and pro/supination are performed while filling a glass with coloured fertilizer and watering the flowers with the matching colour. **b** ‘Chicken run’ addresses transporting, reaching, pushing and retrieval while collecting eggs and bringing them to the egg-cup. Subjects are prompted to collect as much points as possible in a particular time frame while avoiding distractors

I-TRAVLE, stands for ‘Individualised Technology-supported and Robot-assisted Virtual Learning Environment’. It consists of use interfaces for evaluation of arm movements and performance of 3D exercises in a custom-built virtual learning environment. The system has been developed within a European multi-disciplinary cross-border project (Interreg-IV “Rehabilitation Robotics II & I-TRAVLE” IVA-VLANED 1.14; see www.i-travle.eu). This virtual learning environment allows persons to learn and train skill components, which are required during upper limb related activities of daily life. The virtual learning environment enables therapeutic based training of motor function by gradually changing the amplitude, speed, accuracy requirements, and knowledge of performance while providing feedback by the means of haptic, visual and auditory stimuli. The virtual learning environment permits training of different skill components separately such as lifting, transporting, pushing, pulling, reaching and retrieving and rubbing (see Fig. 3 for an illustration). On the other hand, it permits playing of serious games which can be defined as games that are designed for a primary purpose other than of pure entertainment. The developed serious games incorporated simultaneous training of multiple skill components combined with cognitive distractors and challenges. The serious games [28–32] were ‘penguins’ [33]’, ‘arkanoid’, ‘chicken run’ and ‘watering the flowers’ [34] (see Fig. 4 for illustration and description). Each training session consisted of 30 min training, starting with basic motor function exercises of different skill components separately, followed by the serious games [33–36].

All exercises required precise and stabilised end-positions to successfully perform the task-oriented movements. The grasp of objects in the virtual environment was not possible with the hardware (gimbal) given the lack of a gripper, but was enabled when a stable position near the object was maintained for 3 s. The exercises varied regarding number of movement directions (1-2-3D), haptic environment, precision level and type of required movements, cognitive load with distractors and prerequisites for success, etc.

Training in the virtual learning environment was personalized. The initial selection of the basic motor function exercises of different skill components and serious games that was provided to the patient was based on a semi-structured interview. This was inspired on the motor activity log (MAL) on which daily life activities the pwMS wanted to improve [37]. Difficulty levels of the basic motor function exercises of different skill components and serious games were adapted according to the therapist’s clinical judgement on exercise performance and upper limb movement quality as well as the presence of compensatory movements (e.g. trunk flexion, shoulder elevation, etc.). This could be done by changing the training volume, required movement amplitude, the extent of visual, auditory and haptic feedback, the weight of objects and number of distractors. The pre-programmed exercises were semi-autonomously executed by the pwMS but under supervision of the therapist for safety reasons and program adaptation.

### Clinical outcome measures

On function level, the Motricity Index (MI) was applied to measure upper limb muscle strength (pinch grip, elbow flexion and shoulder abduction) with normal score being 100 [38]. Maximal hand grip strength (kg) was determined by means of the JAMAR® hand-held dynamometer (Biometrics Ltd., Ladysmith, USA) [39]. To assess motor control on function and activity level, the Fugl Meyer (FM) was conducted. The FM contains a proximal (items related to shoulder, elbow and forearm movements) and a distal part (wrist movements and grip) with maximal scores of 42 and 24 points respectively and a total maximal score of 66 for the complete upper limb motor section [40].

On activity level, the Action Research Arm test (ARAT) was applied, which assesses the ability to handle objects differing in size, weight and shape as well as gross movements (normal score = 57) [40]. The Motor Activity Log (MAL), Dutch version, was conducted to measure perceived performance of the upper limbs [37, 38, 40, 41]. Participants were asked to score the amount of use (AOU) and quality of movement (QOM) of the upper limbs for 17 pre-defined activities by using an ordinal rating scale (0–5). The MAL USE score is the sum of the AOU and QOM (0–10). Besides, participants’ reports on changes were documented during an interview with open-ended questions. These are questions that cannot be answered with a yes or no but require a comprehensive answer.

### Robotic outcome measures

Participant’s evolution in motor function was also measured in the intervention group with the evaluation-module of I-TRAVLE. This module consists of two parts: the first part measures the active range of motion (aROM) and the second part measures movement duration, velocity and quality (spatial efficiency) during the performance of three skill components.

For the measurement of aROM the participant was instructed to reach out as far as possible into 6 directions (forward, backward, upward, downward, medial and lateral) starting from a standardised starting position with 45° elbow flexion in line with the Haptic Master and the height of the hand at 50 % between the shoulder and knee [30]. In order to guide the movement in the right direction a haptic tube was implemented which restricts deviations in other directions. Reaching distance is expressed in centimetres from starting point till the farthest reaching point for each six directions separately.

Movement duration, velocity and movement quality was measured during reaching (forward and backward directions), lifting (upward and downward directions) and transporting (lateral and medial directions). Participants were instructed to move as fast and accurate as possible. Movement time, shortest distance between the two targets and real covered distance were stored, allowing calculation of the following outcome measures: movement velocity (m/s) was determined as the real covered distance divided by movement time. Movement quality was expressed by the hand path ratio which is the real covered distance divided by the shortest distance between goals, as such reflecting spatial movement efficiency.

### Statistical analysis

The intervention and control group consisted of nine and eight persons respectively. Because of the small sample size, non-parametric statistics were performed. Mann–Whitney U and Chi Square tests compared groups for the descriptive and clinical variables at baseline. The Wilcoxon Signed Rank Test was applied for comparison between week 0 and 8 within each group. For the robotic outcome measures, the Wilcoxon signed rank test was performed between week 0 and 8 for the intervention group only. Statistica software was used with significance level set at p < 0.05.

## Results

Clinical characteristics of the intervention and control group are presented in Table 1. EDSS was overall high (average above 7) indicating inclusion of pwMS with high overall disability with most of the pwMS being wheelchair bound. The majority of pwMS predominantly used their right arm in daily life, which was also the trained arm in approximately half of the intervention group.

**Table 1:** Clinical characteristics of the MS intervention and MS control group

Subject	EDSS	Age	Sex (F/M)	Type of MS	Disease duration (yrs)	Wheelchair-bound	Current hand dominance	Arm trained
MS Experimental group								
1	8.5	65	F	SP	25	Yes	Right	Right
2	8.0	57	M	SP	27	Yes	Right	Left
3	8.0	75	M	SP	10	Yes	Right	Right
4	8.0	64	M	PP	14	Yes	Left	Left
5	8.0	56	M	SP	26	Yes	Right	Right
6	8.5	55	M	SP	27	Yes	Right	Left
7	3.5	58	F	SP	3	No	Right	Left
8	8.0	72	M	SP	34	Yes	Left	Left
9	7.0	47	F	RR	24	Yes	Right	Left
Median	8	58			25			
IQR	8-8	56–65			14–27			
MS Control group								
1	7.0	59	M	SP	18	Yes	Left	Right
2	7.5	46	F	SP	10	Yes	Right	Left
3	7.0	50	M	SP	8	Yes	Right	Left
4	8.0	52	F	SP	14	Yes	Right	Right
5	8.0	49	F	SP	23	Yes	Right	Right
6	3.5	48	F	SP	5	No	Right	Left
7	6.5	62	M	SP	21	No	Right	Left
8	7.5	59	M	PP	/	No	Right	Right
Median	7.3	61			14			
IQR	6.9–7.6	47–75			9–19.5			
p	*0.25* ^*^*^	*0.11* ^*$*^			*0.09* ^*$*^			

Values reported are median and interquartile range, or number; *p*= p-value chi square test for nominal/ ordinal data or Mann-Whitney U Test for continous data to compare groups *yrs* years, *F* female, *M* male, *SP* secondary progressive MS, *RR* relapsing remitting MS; *PP* primary progressive MS

At baseline, the intervention and the control group were not significantly different regarding type of MS, EDSS, disease duration and all clinical and robotic upper limb outcome measures. After 8 weeks training, a near-threshold significant difference (*p* = 0.048) was present between both groups for the MAL amount of use and the total MAL.

**Fig. 5:**
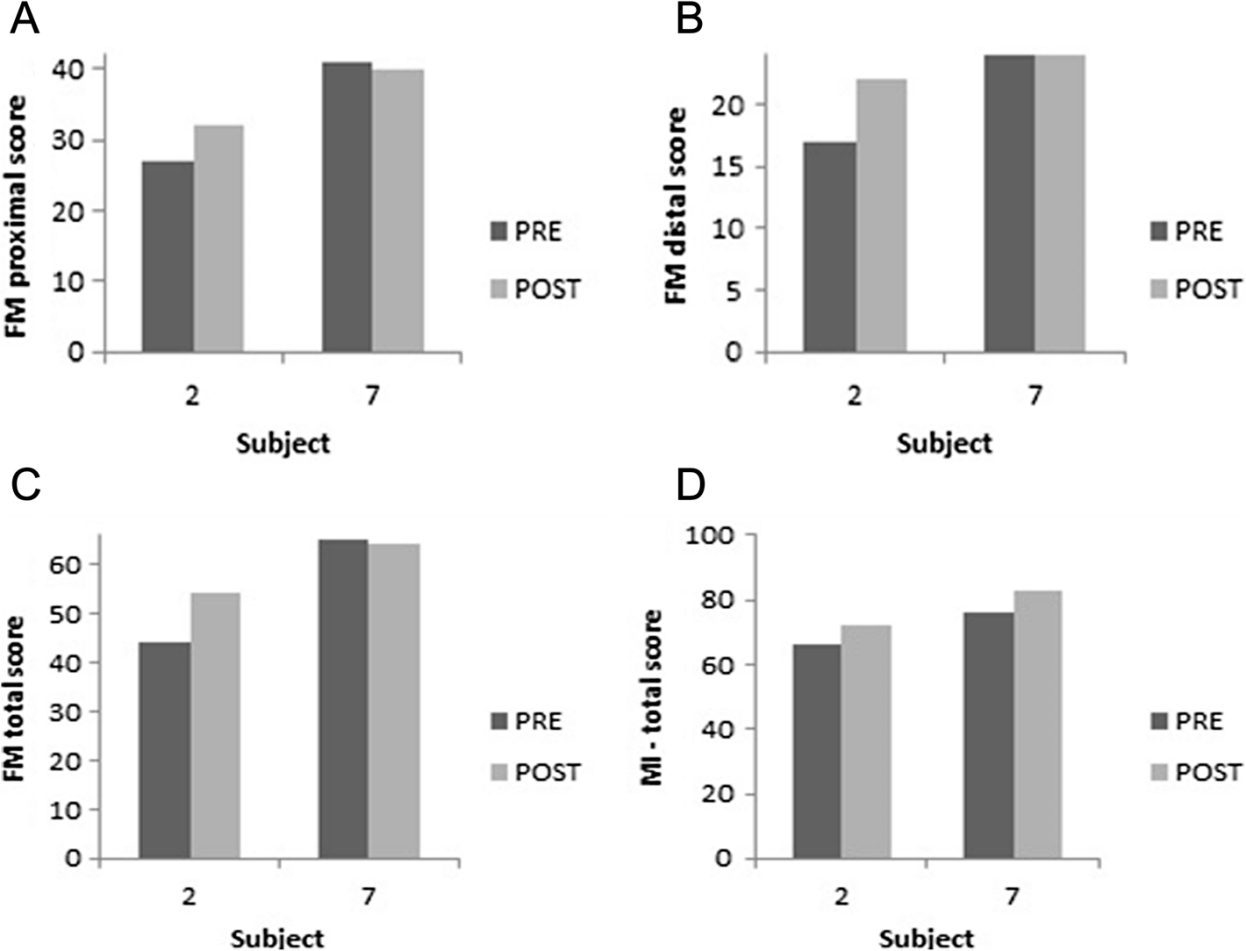
Proximal (**a**), distal (**b**) and total (**c**) FM score and Motricity Index (**d**) for subjects 2 and 7, pre- and post-I-TRAVLE training

There were no significant changes found over time in neither the intervention nor the control group (Table 2). Observation of the raw data indicated that five persons in the intervention group, with marked to severe arm dysfunction improved on the Motricity Index and/or the Fugl Meyer test. Figure 5 illustrates the changes on the FM and MI for subject 2 which is representative for pwMS with marked to severe arm dysfunction and subject 7 which is representative for pwMS with a mild upper limb function according to the scores on FM and MI.

**Table 2:** Experimental clinical outcome measures (median [interquartile range]) at baseline and 8 weeks for both the intervention and control group

		Intervention group (*n* = 9)				Control group (*n* = 8)			
		Pre	Post	∆	p	Pre	Post	∆	p
Motricity Index, 0–100		72.0 [59.0–76.0]	64.0 [60.0–83.0]	−8	ns	66.0 [50.8–70.5]	59.5 [46.3–77.8]	−6.5	ns
Hand grip strength (kg)		21.3 [12.0–23.3]	21.0 [10.7–23.3]	−0.3	ns	16.3 [11.7–19.5]	17.0 [11.3–19.0]	0.7	ns
Fugl Meyer, 0–66		52.0 [43.0–62.0]	52.0 [43.0–63.0]	0	ns	55.0 [40.0–57.5]	56.0 [40.0–59.0]	1	ns
	Distal score, 0–24	20.0 [17.0–23.0]	19.0 [18.0–23.0]	−1	ns	19.5 [16.5–21.8]	20.0 [16.8–21.8]	0.5	ns
	Proximal score, 0–42	33.0 [27.0–38.0]	32.0 [25.0–39.0]	−1	ns	33.0 [25.0–36.5]	34.0 [25.0–36.5]	1	ns
Action Research Arm test , 0–57		40.0 [20.0–41.0]	38.0 [27.0–47.0]	−2	ns	36.0 [28.5–41.0]	35.0 [27.5–44.0]	−1	ns
Motor Activity Log, 0–10		5.3 [2.1–8]	5.2 [4.3–7.1]	−0.1	ns	2.1 [1.5–4.7]	2.0 [0.7–5.1]	−0.1	ns
	Amount of use, 0-5	3.0 [1.2–3.8]	3.0 [2.5–3.5]	0	ns	1.2 [0.9–2.5]	1.0 [0.5–2.6]	−0.2	ns
	Quality of movement, 0-5	2.3 [1.0–4.0]	2.4 [1.8–2.9]	0.1	ns	0.9 [0.7–2.1]	0.9 [0.3–2.4]	0	ns

Values reported are median and [interquartile range]; *p* = p-values, Wilcoxon Signed Rank Test. For all outcome measures, a higher score indicates a better upper limb performance. A positive delta indicates an improvement of the score after training compared to baseline and vice versa for a negative sign

**Fig. 6:**
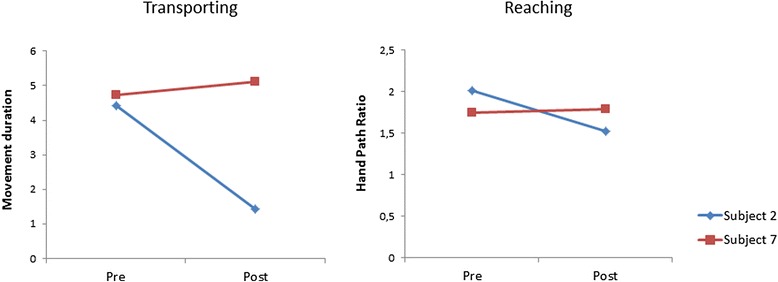
Movement duration of transporting exercise and hand path ratio of the reaching exercise for subjects 2 and 7, pre- and post- I-TRAVLE training. Movement duration is expressed in seconds while the Hand Path Ratio is the real distance covered between the two goals divided by the shortest distance between the goals

Table 3 provides the results of the robotic generated outcome measures after 8 week training of the intervention group, providing information on active range of movement as well as movement speed and spatial accuracy in three dimensions. It appeared that active range of movement in the different directions remained unchanged. This result may perhaps be related to the limitations in the workspace of the haptic master itself, which was maximal for part of the pwMS at baseline. The intervention group, at group level, increased overall velocity during reaching, lifting and transporting, and thus reduced movement duration for all skill components. Movements were also more efficient in the spatial domain as revealed by a decreased hand-path ratio for transporting and some trend towards significance for reaching. Lifting movements did not change in the spatial domain. Figure 6 illustratively shows individual changes in movement duration during transporting and hand path ratio during reaching for subject 2 and 7, before and after I-TRAVLE training.

**Table 3:** Experimental robotic outcome measures in the MS intervention group during transporting, reaching and lifting

		Median Pre [Q_1_–Q_3_]	Median Post [Q_1_–Q_3_]	∆	*p-value*
Transporting (Left-Right)	ROM (m)	0.666 [0.512–0.709]	0.622 [0.513–0.745]	−0.044	ns
	Duration (s)	8.459 [5.955–21.324]	6.853 [5.957–7.044]	−1.606	0.043*
	Speed (m/s)	0.163 [0.146–0.170]	0.206 [0.177–0.237]	0.043	0.018*
	HPR	1.554 [1.388–3.413]	1.088 [0.978–1.451]	−0.466	0.017*
	Distance (m)	1.304 [0.904–2.878]	1.307 [1.196–1.382]	0.003	ns
Reaching (Front-Back)	ROM (m)	0.375 [0.302–0.406]	0.405 [0.314–0.407]	0.030	ns
	Duration (s)	7.575 [4.372–12.540]	5.802 [4.106–6.842]	−1.773	0.063**
	Speed (m/s)	0.103 [0.072–0.175]	0.146 [0.120–0.177]	0.043	ns
	HPR	2.114 [1.850–2.708]	1.894 [1.877–2.300]	−0.220	0.091**
	Distance (m)	0.782 [0.687–0.936]	0.848 [0.656–0.905]	0.066	ns
Lifting (Up-Down)	ROM (m)	0.436 [0.436–0.436]	0.436 [0.411–0.436]	0.000	ns
	Duration (s)	9.137 [6.141–10.804]	6.319 [3.268–9.106]	−2.818	0.008*
	Speed (m/s)	0.128 [0.083–0.167]	0.152 [0.104–0.214]	0.024	0.051**
	HPR	1.639 [1.572–2.863]	1.668 [1.448–2.244]	0.029	ns
	Distance (m)	0.810 [0.776–1.414]	0.810 [0.715–1.032]	0.000	ns

Values reported are median and [interquartile range]

*HPR* hand path ratio, *m* meters, *s* seconds, *m/s* meter per second, *ns* not significant

^*^significant Wilcoxon Signed Rank Test < 0.05; ^**^ trend towards significance 0.05 < p < 0.1

PwMS in the intervention group spontaneously reported beneficial changes in the daily use of their upper limb(s). One pwMS mentioned the obtained ability to eat a complete meal independently instead of only some initial independent bites (previously requiring the help of a nurse), as well as the ability to write longer emails. Another pwMS mentioned the ability of bilateral arm use such as simultaneously driving an electrical wheelchair with one hand while scratching their nose with the other hand; instead of doing those activities sequentially. Another pwMS reported the ability of hugging his wife again with both arms, which was not possible previously.

## Discussion

The present pilot RCT reported on changes in (perceived) upper limb function, in highly disabled pwMS with marked muscle weakness, after 8 weeks of additional robot-supported I-TRAVLE upper limb training compared to conventional treatment only. Effects were found in favor of improved motor control captured by the robot evaluation application and were mirrored by testimonials indicated on beneficial and meaningful effects in daily life. In contrast, no effects on standard clinical outcome measures on function or activity level of the ICF were present on group level.

It was observed that pwMS can improve motor control after robot-supported therapy in a virtual learning environment. While previous pilot work in MS measured changed kinematics during two-dimensional horizontal reaching [18–20], changes in the present study were present in three different directions (reaching, transporting, lifting) for movement speed. Furthermore, improvements were present in the spatial domain with increased movement efficiency during transporting, and indications for reaching. A reduced hand path ratio indicated that pwMS moved close to the optimal trajectory between the starting position of the hand and the target. These findings might suggest that there is a restorative potential of upper limb motor function in pwMS with overall high disability. This suggestion is important given that MS is a chronic and overall progressive disease, being characterized by high lesion load and/or brain atrophy in the more disabled pwMS [42]. A preserved motor learning potential had been demonstrated before in pwMS despite a high burden of cerebral pathology, providing a neuroscientific rationale for recovery-oriented strategies [43, 44]. One may however argue that improvements in motor function were related to adaptation of the pwMS towards the inertia of the haptic master, during the eight weeks of training. Although the latter is conceivable, it is pointed out that the target locations were not identical those provided during the training program. We do believe that upper limb movement control has really changed in the intervention group given the testimonials on very specific and upper limb related improvements in daily life. However, in future research it is recommended to use other sensor or robotic devices than the actual training device to evaluate movement quality improvements after rehabilitation.

There were no significant group effects on the clinical measures which was rather unexpected given that the robot-supported I-TRAVLE training was focused on motor function and goal-oriented movements, and applied according to therapeutic principles regarding training load and motor learning [9]. Different factors may be discussed to explain the negative findings on group level, in contrast to beneficial effects in individual cases. First, the training may have been focusing mainly on proximal upper limb movement, which appeared to improve the robotic motor control tests but are not immediately translated to the tests encompassing distal hand function. Previous pilot studies in MS have indicated the possibility to improve upper limb function use after technology-supported training [20, 45]. These studies applied a three dimensional electromechanical device including a handle for grasp function to be controlled to successfully execute virtual games, and a one-dimensional robot including a condition where real-life objects had to be manipulated during training. The presented I-TRAVLE set-up allowed finger movements during movement execution which were however not required for the goal-directed movements in the virtual learning environment, and therefore likely not often performed. As such, no impact of I-TRAVLE training was found on the ARAT which requires manual dexterity for object manipulation. Maximaluscle strength was not increased after training. This was expected as the present intervention focused on motor control without including components of resistance training. The combination of unchanged muscle strength and manual dexterity may make it logical that also the MAL, reflecting perceived upper limb use, was not changed in contrast to previous technology-supported studies including a grasp function in MS and stroke [41, 46–49]. It is advised that technology-supported training should encompass a hand grip component in order to expect direct effects on manipulative tasks which are often required in daily life [50].

Positively, the applied robot-supported intervention was overall experienced as motivational by all pwMS indicating the feasibility of applying haptic robots and virtual learning environments in clinical practice. With the lack of effects on group level, it seemed that it was too early to already apply a randomized controlled design [51]. It is important to identify those pwMS that showed improvement on standard clinical tests. Similarly, it has to be investigated which improvement in daily life was experienced-perhaps even beyond their expectations-given their already long-standing chronic condition. Unfortunately, current sample size did not allow for statistical analyses in subgroups. Observation of the raw data indicated that especially pwMS with marked- to-severe upper limb function showed considerable improvement on the included clinical tests. As such, the robot-supported therapy may be most suitable for low-functioning pwMS, while high-functioning pwMS would benefit from other interventions encompassing hand function [52, 53]. In this context, it is important to include qualitative measures on perception. Case testimonials especially from pwMS with marked to severe upper limb dysfunction indicated that some pwMS were able to perform (simultaneous bilateral) movements with less efforts and for a longer time, as reflected in the stories on continued wheelchair driving, full meal eating and email writing. These testimonials may suggest that robot-supported training, including many active movement repetitions within one training session, improves muscle endurance and therefore capacity to continue functional movements longer. Future research should therefore consider the inclusion of objective outcome measures regarding muscle endurance. It is also advised to add accelerometry for the measurement of the actual upper limb performance in daily life [49]. It is conceivable that improved muscle endurance leads to more intensive use of the upper limbs in daily life despite that there was no improvement in maximal muscle strength. Daily life use of the upper limb does also relate to fine motor dexterity [49]. Unfortunately, also impeding comparison of effects between studies, we had not included the Nine Hole Peg Test in our test battery as our robot-supported training did not include hand movements. It is acknowledged that previous research has shown the possibility of carry-over of proximal arm training to distal hand function [16]. Finally, we have observed different responses to our treatment. In order to better identify responders to the training intervention, details of the training content, volume and intensity level should be documented in more detail in future and well-powered studies. As well, addition of neuro-imaging would allow to better understand whether changes are related to training counteracting disuse or to structural neuroplasticity.

The present study was executed with a custom-built I-TRAVLE system that is still in further development. It was mentioned above that an interactive hand module should optimally be added to incorporate hand function during serious gaming. Further recommendations are related to training intensity. Perhaps the 30 min of training per session, which is including start-up and navigation through the virtual learning environment between games, was too short. Moreover, out of protectiveness towards pwMS, the increase of difficulty levels by the therapist may have been too slow. Therefore, our research is currently addressing adaptive systems which would also support full autonomous training with I-TRAVLE [33, 35]. The HapticMaster, with its potential to provide anti-gravity support, proprioceptive feedback on the optimal trajectory as well as of the virtual environment, allows for sensory integration. Which is believed to resemble real life to a larger extent than pure visual virtual environments. However, the robot-assisted approach with haptic and visual feedback may be mostly indicated for pwMS with severe arm dysfunction who are yet unable to perform successfully activities in real life. Other interfacing technologies, of which lightweight sensor-based technologies and camera-based approaches are well-known examples, may be more appropriate for pwMS with high functioning upper limbs.

## Conclusion

Robot-supported training in a personalized virtual learning environment was feasible and lead to significant changes in motor function in highly disabled pwMS. However, this was on group level, not reflected by significant changes on standard clinical tests, which include hand function. Persons with marked upper limb dysfunction may clinically benefit most from additional robot-supported training. Larger samples are needed to further investigate the optimal training dosage and modalities in order to maximize effects.
